# Enhanced layered fog architecture for IoT sensing and actuation as a service

**DOI:** 10.1038/s41598-021-00926-y

**Published:** 2021-11-04

**Authors:** Abdulsalam Alammari, Salman Abdul Moiz, Atul Negi

**Affiliations:** School of Computer and Information Sciences, University of Hyderabad, Hyderabad, 500046 India

**Keywords:** Computational science, Computer science, Information technology, Mathematics and computing

## Abstract

The reduced service cost offered by Sensing and Actuation as a Service paradigm, particularly in Internet of Things (IoT) era, has encouraged many establishments to start without worrying about having their own infrastructure. Such a paradigm is typically managed by a centralized cloud service provider. Fog paradigm has emerged as a mini-cloud that if designed with care to assist the cloud, together will achieve better performance. This article introduces a layered fog architecture called Sensors and Actuator Layered Fog Services Delivery (SALFSD) for IoT ecosystems. The significance of SALFSD is being fault resistant; it dynamically reassigns tasks of the failed node to the nearest active node to maintain the network connection. Besides, SALFSD monitors end users pre-specified cases closer to the physical devices hired by end users to fasten generating the actuation commands. Such node may offload its monitoring responsibility to its parent node in case it is overloaded. SALFSD is evaluated using Yet Another Fog Simulator in different scenarios (numbers of users, sensors, actuators, and areas). A comparison was made for Sensing and Actuating as a Service (SAaaS) with/without layered fog, and layered fog with/without (failure reassignment, pre-specified cases in fog nodes, and offloading). The comparison was conducted in terms of computing/communication latencies and the number of missed messages for both observations and actuation commands. Results show that failure reassignment prevented losing messages and maintained network connectivity. Also, wisely selecting the monitoring fog node per end user pre-specified cases and the offloading scheme decreased actuation latency.

## Introduction

Sensing and Actuating as a service (SAaaS) is a paradigm where several types of sensors and actuators (Ss/As) -stand-alone or in wireless sensor networks- are provided to the end users as per their desired composition upon which end users can build their application. Typically, SAaaS infrastructure is governed through cloud computing mechanism; device owners offer the functionality of their devices to the service provider either free of cost or on rent bases. While the cloud act as an intermediate interface which delivers the system’s resources as services for the registered users as agreed in the purchased offer. Thereby, the users will be charged on the bases of a pay-as-you-go pricing model which permit the users to scale the system’s resources easily.

SAaaS is mainly a complex paradigm. Besides that, the system must be able to operate under a diversified ecosystem and properly manages the observations and actuation requests. Also, the infrastructure needs to be highly virtualized to permit the exploitation of the same physical layers components by multiple users simultaneously, which can reduce the cost of the service^2^.

Existing literature in SAaaS has exploited fog computing to process part of IoT and cloud tasks closer geographically to data sources rather than fully relying on the remote cloud datacenter. However, fog nodes may be overloaded due to the amount of sensing data generated by IoT sensors leading to processing delay. In this work, to avoid actuation commands generation delay, we monitor the users’ specified cases in the fog layer and allow each fog node to offload the monitoring to its parent once it is overloaded.

To this end, a scalable and failure resistance layered fog architecture is proposed for SAaaS paradigm. The main objective of this work is to enhance the performance of SAaaS for IoT paradigm by introducing fault resistant layers of fog nodes in between the cloud and IoT devices. We looked at such an idea of adding fog layered from different angle comparing the typical use of fog node (i.e. data filtering before sending to the cloud). This work aims at including more tasks in fog nodes that are expected to enhance the overall performance of the service provider while keeping in mind the capability of fog nodes as being less featured compared with the cloud.

### Contribution

To the best of our knowledge, this work is the first attempt to have multiple layers of fog nodes in between the cloud and IoT devices in SAaaS with the main contributions listed below:

1. Failure plan: as every fog node is known to be a point of failure in the network, a dynamic reactive failure plan is proposed to maintain the connectivity by reassigning the tasks of the failed fog node to the nearest connected fog node in the same layer or to the parent of the failed node.
2. Specified cases monitoring (SCM): typically, in SAaaS, end user receives the observations and decides upon the desired actuation to be triggered. However, end user may have pre-specified actions to be triggered in response to some types of sensor(s) observations thresholds. We deploy the monitoring of such cases in the fog nodes as well as in the cloud.
3. Monitoring offloading: the default fog node responsible for monitoring the user specified cases is the one closer to the gateway(s) connected to Ss/As rented by the end user. However, as the specified cases needs actions to be taken fast, we may dynamically handle the monitoring task to the parent of the fog node in case it is overloaded to reduce the actuation latency.
4. Observations filtering: fog nodes also filter the observations before forwarding and drop the corrupted messages - if any- to avoid unnecessary bandwidth consumption.
5. Sensors and actuators selection: we have implemented the Nondominated Sorting Genetic Algorithm II (NSGA-II)^7^ to select the optimal Ss/As of end user interest types at their chosen location. The selection is bi-objective which is based on cost and feedback.

**Figure 1:**
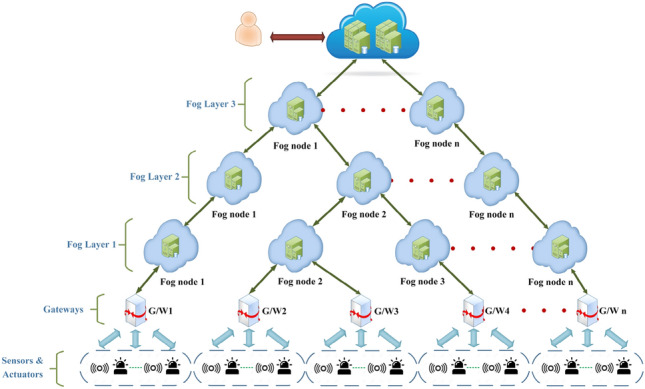
SALFSD topology.

### Example scenario

Assume that SALFSD service provider has set up its infrastructure which covers a particular geographical area; city(s), country(s), or region(s) as shown in Fig. 1. The service provider has agreements with many Ss/As devices owners (mobile devices, wireless sensor networks, or even standalone devices) that are linked to gateways. The gateways are connected to fog nodes in the lowermost fog layer in SALFSD infrastructure. As per the agreement, sensor devices must send their observation to the services provider frequently/continuously as per the time agreed. Also, actuation devices owners must allow the service provider to send actuation commands to be executed.

Now assume end user (environment specialist, agriculture monitoring agency, forest monitoring, etc.) is interested in getting observations of type(s) of sensors either coming under one or several networks/areas. According to the analysis of the received observations, end user may decide to send actuation command(s) to one or many actuators. Again, actuators may be under one or several networks/areas. As explained in contribution subsection, SALFSD deploys SCM of each end user -if any- in fog nodes as well as in the cloud where the said node will trigger the actuation command on behalf of the end user.

Following is the organization of remaining sections in the article. “Related work” Section summarizes the related work in SAaaS. The proposed approach is demonstrated in “Proposed architecture” section. “Experiment methodology and results discussion” section presents the experiment methodology and results discussion. This is followed by formal verification in “Formal verification of architecture correctness” section. Finally, the article is concluded in “Conclusion and future work” section.

## Related work

This section reviews SAaaS works and issues addressed in the literature. Some works proposed either a stand-alone architecture for SAaaS or extended an existing framework. While others have implemented their proposal inside a reference architecture with valuable contribution attempting to solve general IoT and SAaaS related issues.

The main building blocks and players of SAaaS were identified in^3^. The work also introduced an architecture for SAaaS with a hypervisor as an important component that operates at sensors’ nodes level. Based on control policies set by the cloud modules, the hypervisor provides virtualization functionalities for the physical devices and handles the interaction between different types of devices.

A more detailed SAaaS architecture based on^3^ was introduced in^4^ were abstraction and virtualization of physical resources were addressed, allowing composition of several instances out of the same resources. A further extension in^5^ brought about a utility framework for SAaaS inside IoT-A reference architecture. The same was implemented for a real IoT scenario.

Another SAaaS framework was proposed in^2^ called Stack4Things that extend the cloud’s OpenStack resource management framework using IoTronic and Ceilometer services. Through its core, the IoTronic service manage the databases containing the IoT devices specifications such as their identifiers and the associate user. The Ceilometer service is responsible for sensing data collection and storing it in a non-SQL database; hence, the users can interact with this database to obtain the requested events. In IoT-client side, the board is equipped with several modules to gather sensors’ generated data and permit communication with the cloud services.

Further,^6^ proposed a novel model of system management and computing for SAaaS called Sensing and Actuation as a Service Delivery Model (SAaaSDM) which is based on a cloud edge centric IoT model. The purpose of SAaaSDM is to overcome the issues of centralized cloud architecture while taking advantage of computing and storing capacities of both cloud and edge computing. Compared with other works, SAaaSDM brings many benefits to sensing and actuation applications. In addition to exploiting virtualization and enabling collaboration between various type of IoT devices, SAaaSDM enhances the performance of the paradigm by decreasing both bandwidth usage and processing response time as well as improving the network lifespan.

The issue of heterogeneity and interoperability of IoT devices were tackled in^1^. Authors designed an IoT platform (nCube) based on oneM2M standard, which unifies the communication language between IoT devices. To achieve such unified communication, they developed middleware programs (thing adaptation software TAS) which enable the translation of sensing value and actuation commands into a defined oneM2M resources accessible and manageable through a web application.

Most recent works in^12,13^ authors worked towards achieving parallel processing of IoT tasks by applying horizontal computation offloading. The task is received to the fog node (initially selected in a greedy way.) This fog node applies a horizontal task distribution of the independent subtasks by forming Directed Acyclic Task Graph (DATG). For parallel execution, the subtasks are then offloaded to other fog nodes on the same level. These works also considered the dynamicity of the network and made a reputation manager in each fog node to monitor the nearby node that offloaded the task. The works have reduced the operation delay and energy consumption comparing to traditional fog and cloud computing.

Below we present feature-wise comparison for our proposed work against the aforementioned related work with respect to points listed in Table 1.

**Table 1:** Comparison of proposed work with related work.

References	^3^	^4^	^5^	^2^	^6^	^12,13^	SALFSD
Topology	Node-cloud	Node-cloud	Device-cloud	Node-cloud	Cloud edge-centric	IoT-fog	G/W-layered Fog-cloud
Processing at	No *	Mote, end user	Mote, end user	Cloud	edge, cloud	Fog layer	Fog, cloud
Virtualization	Device-level	Device-level	Device-level	At cloud (OpenStack based)	At cloud	No	At cloud
Failure plan	No**	No**	No**	No**	No	Yes***	Yes
SCM	No**	No**	No**	No**	No	No	Yes
Monitoring offloading	No**	No**	No**	No**	No	No***	Yes
Observation filtering	No**	No**	No**	No**	No	No	Yes
Working model/simulation	No	No	Only For Android	No	No	Yes	Simulated by YAFS^10^

Regarding processing in^3^ (*), the work did not state any thing about data flow or processing. Also with respect to failure plan (**), works^2–5^ do not adopt any concept like Fog or Edge nodes which is where SALFSD has the failure plan, specified cases monitoring, monitoring offloading, and observation Filtering.

Regarding failure plan (***) in^12,13^ the monitor works as a watchdog that observes the nature of the devices offering the services within the RAN. If the node fails, the tasks or subtasks are reassigned to another fog node. Also, they assume that the fog nodes don’t fail during the execution, while we assume the failure to occur at any time during the execution.

With respect to monitoring offloading (***),^12,13^ works are based on the horizontal distribution that offloads the data for parallel processing. Our offloading contribution in the designed architecture is meant of offloading the monitoring of the specified case. In this case, the nodes in the same level don’t have the information required to monitor the specified cases; information is available with the dedicated node for monitoring, its parents, and the cloud. Hence, the offloading can happen only to the upper layer -to the parent node only. And this offloading occurs only in case the dedicated node for monitoring is overloaded.

This comparison is based on features (listed in the table) which highlights the main differences among works involved in the comparison.

While studying issues and architectures proposed in related work, we noticed that fog computing had not been exploited yet to work with the cloud in SAaaS. As fog computing can take a load from the cloud and also enhance the overall SAaaS performance particularly if few important tasks are being executed in fog nodes as per their geographical location, we reach to the idea of layered fog architecture.

## Proposed architecture

The proposed architecture in this work is an extension of our previous published work^8^ where we theoretically presented a high-level design of SALFSD discussed our expectations of SAaaA performance enhancement considering SALFSD design. Besides, this work details entire SALFSD components and communication among them, simulates, and adds some improvements to^8^ by considering the key performance metrics of SAaaS from network and architecture points of view.

Mainly, SALFSD is a three layers architecture; IoT layer, fog layer, and the cloud. SALFSD aims at enhancing the performance of the typical SAaaS paradigm with several layers of fog nodes between the cloud and the sensing and actuation layer (IoT layer).

SALFSD is designed to contain several layers of fog nodes; the number of layers and their deployment depends on the organisation of the geographical area covered by the service provider and has no technical impact on the design. For instance, it can be as organisations, cities, countries and so on as depicted in Fig. 1. IoT devices are connected to the gateways connected to fog nodes in the lowermost fog layer, which in turn are connected to the upper fog layer. The uppermost fog layer is connected to the cloud. There is no collaboration or connection among the fog nodes in the same layer; all go through the cloud.

To this end, the performance metrics governed SALFSD design are: (1) Scalability and availability of SAaaS provider network. (2) Avoiding observations and actuation commands loos due to fog node(s) failure while maintaining reduced observation and actuation latency.

Below we explain SALFSD layers, their components and communication among them with details.

### SALFSD cloud

**Figure 2:**
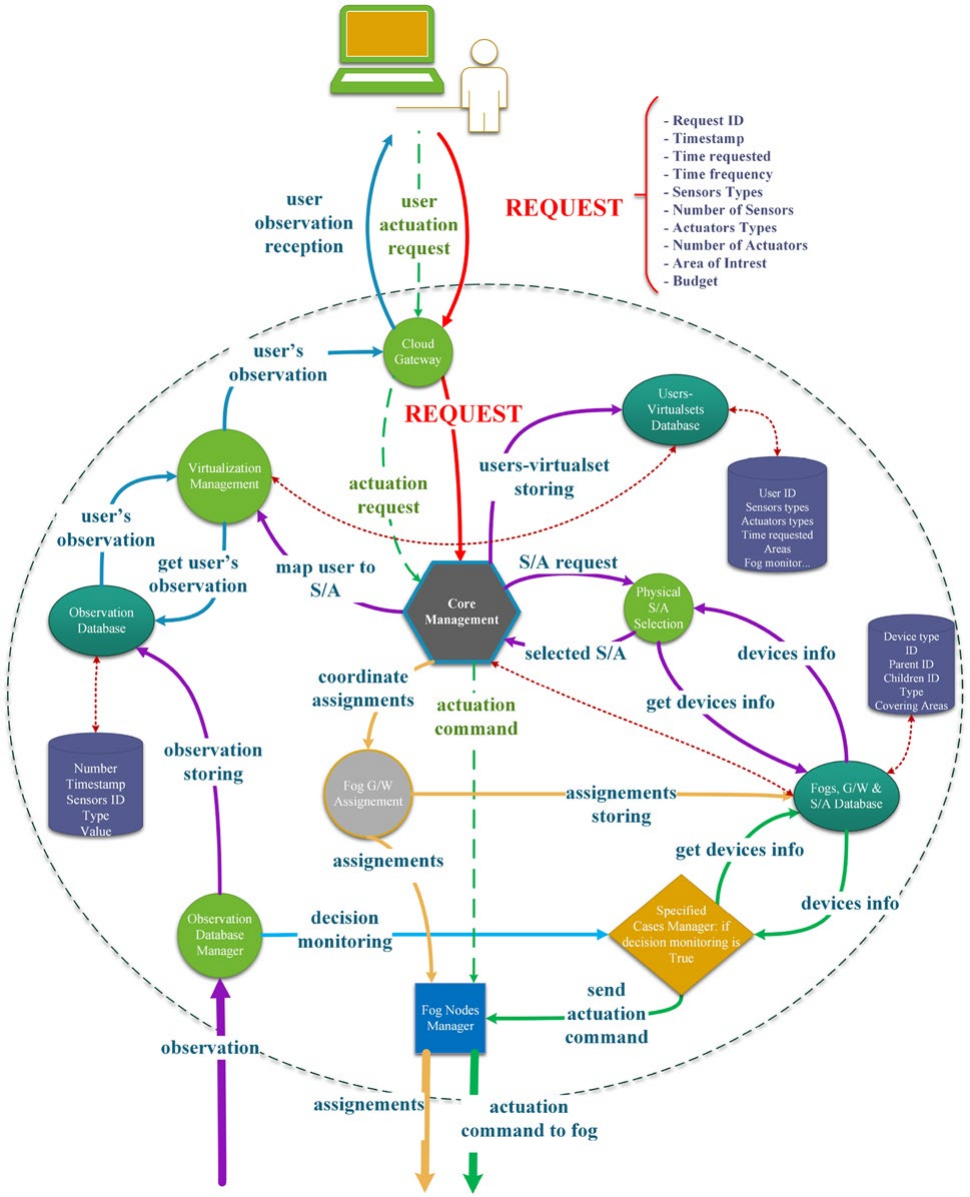
Cloud design with all its components and types of internal messages among its models.

This subsection details the components of SALFSD cloud and their responsibilities as depicted in Fig. 2. The communication among the cloud, fog and gateway layers is depicted in Fig. 19.

#### Cloud gateway

Cloud gateway is the interface with the end user for registration, receiving requests (required Ss/As and other details), sending observations to end user, and receiving end user’s actuation requests. End user’s requests are forwarded to Core Management.

#### Core management

Core management extracts types and numbers of Ss/As requested by end user and budget, sends them to Physical S/A Selection model to get the optimal S/A devices of types requested by end user in his area of interest. Virtualization Management model is informed about those selected devices.

#### Physical S/A selection

Physical S/A selection is responsible for selecting the optimal Ss/As of types requested by end user in their area of interest, the selected Ss/As are passed to Virtualization Management.

End user may request set of Ss/As which may be available with different feedback ratings and different associated costs. For effective based service, there is a need for bi-optimization of Ss/As cost and feedback as below:

1 $$\begin{aligned}&Minimize (F_{Cost}) \end{aligned}$$

2 $$\begin{aligned}&Maximize (F_{Feedback}) \end{aligned}$$

NSGA-II is a well known multi-objective optimization algorithm and has solved several optimization problems in IoT and many other different areas.

SALFSD strategy adapted NSGA-II method where every task is defined as vector to be optimized. Comparison of cost and feedback values produces an optimal Ss/As list. Algo. 1 shows SALFSD NSGA-II based algorithm. The solution list, generation number and Ss/As list are Initialized (lines 2–4). Then the list of active Ss/As are aggregated and assigned to solution list(lines 5–8). Each solution is equipped with fitness_1 and fitness_2 functions (lines 9–11). In order to equate each population sample values with the other solution from the list, NSGA-II uses a rapid $non\_dominated\_sort()$ to filter the individual solution list into some kind of different dominant types (line 12). Then crowding distance is calculated to find nearby values (lines 14–15). $Crossover\_Mutation()$ is used to produce the new population (lines 17–18). As per the non-dominated strategy, every entity governs another, even if the dominant term is utilized to break the N-list into a number of fronts (lines 20–23). SALFSD utilizes $crowding\_distance()$ to choose a subset of single solutions of similar domination rank by removing the fronts (lines 24–25). Such phases go on until the end criterion for completion is met. Then the optimal top-N suggestions for specific solutions are considered. The maximal optimum $S/A\_id$ will therefore be selected (lines 27–34).

3 $$\begin{aligned}&CD_{im}=\frac{f_{m}(x_{i+1}) - f_{m}(x_{i-1})}{f_{m}(x_{max}) - f_{m}(x_{min})}, i=2 \ldots ,(l-1) \end{aligned}$$

4 $$\begin{aligned}&CD_{i}=\displaystyle \sum \limits _{m=1}^M CD_{im} \end{aligned}$$

The crowding distance is calculated as per Eqs. 3–5. In Eq. 3, in ascent order of fm, sort and compute all the ’l’ solution in a pareto front and in Eqn. 4, reiterate it for every objective and identify the crowding distance of i solution. In Eq. 5, owing to two i and j solutions, the solution i would prefer to j as it is with lower (better) rank than solution j or both may be with the same rank but solution i is in less crowded region.

5 $$\begin{aligned} Ri< Rj\ or\ ( Ri=Rj\ and\ CDi >CDj) \end{aligned}$$

Algo. 2 selects the optimal $Ss/As\_id$ based on 3 cases of end user budget inputs: (1) If there are Ss/As in *S*/*A* equal to the given budget, then the specific optimal Ss/As Id(s) will be selected (lines 5–6). Else, (2) nearest cost to end user budget in the list of *S*/*A* with a certain threshold value are considered for the optimal $S/A\_id(s)$ selection (lines 7–8). (3) If budget is not taken into account, all available *S*/*A* are considered for the optimal selection (lines 9–11).

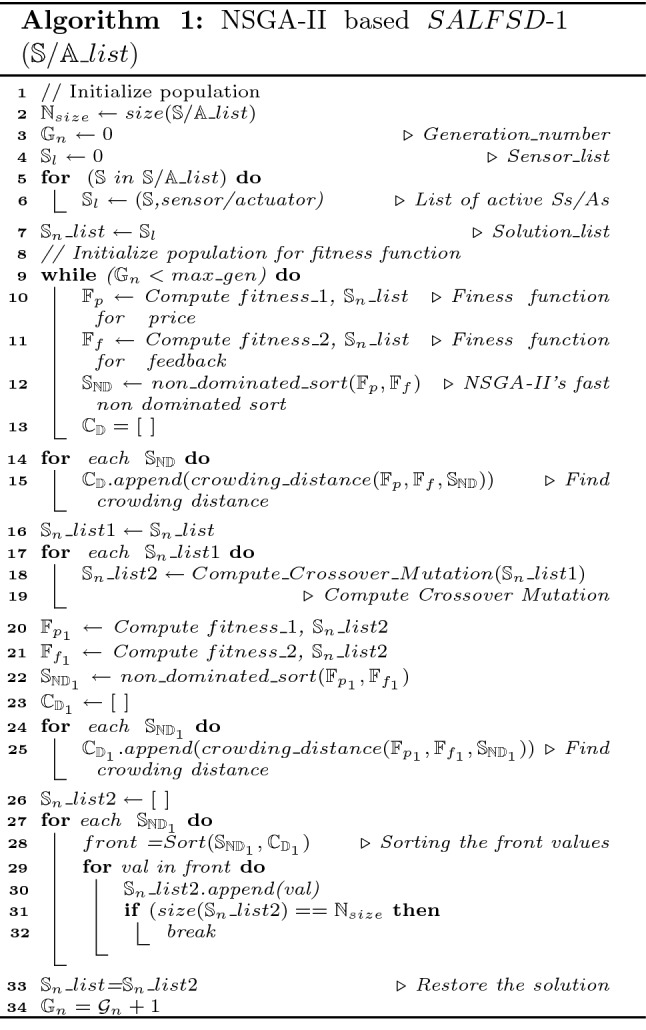

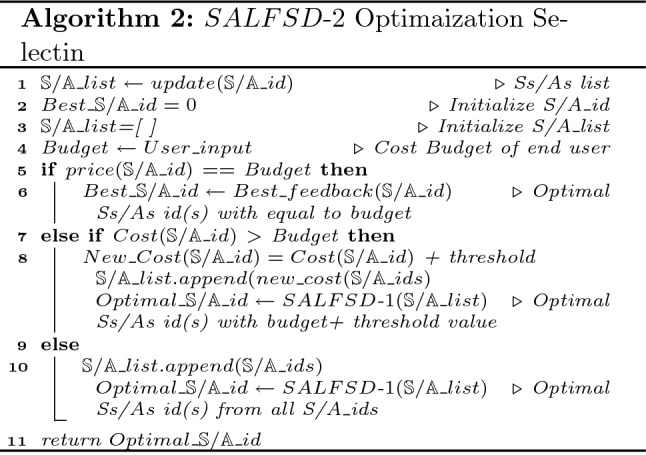





#### Virtualization management

**Figure 3:**
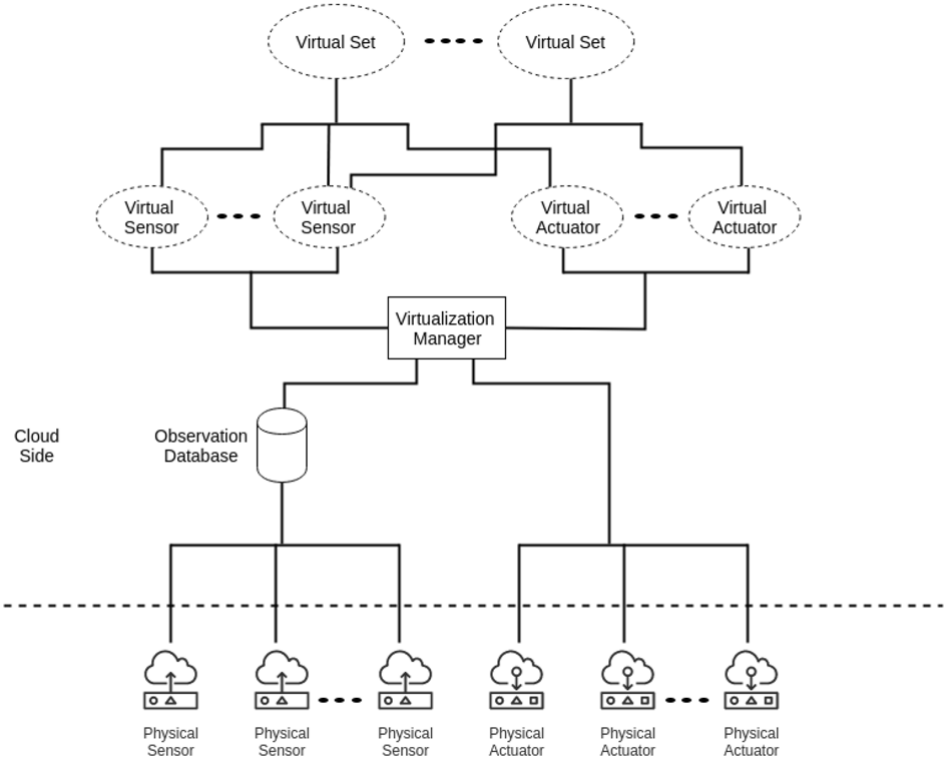
Virtualizing sensors and actuators.

Once the optimal Ss/As are selected, the virtualization manager is in charge of creating virtual sets for each user as per their selected Ss/As. the virtualization manager is also responsible for handling sensors observations to the cloud gateway to be sent to the end user. Fig. 3 shows Ss/As virtualization.

#### Fog G/W assignment

This model takes care of assigning end user specified cases monitoring to the nearest fog node as per their geographical distribution. For example, in Fig. 1, suppose that end user1 has hired Ss/As in G/W1, then fog node1 in fog layer1 will be assigned the monitoring of end user1 specified cases. If end user has hired Ss/As from G/W2 and G/W4, then fog node2 in fog layer2 will be assigned the monitoring. In case end user Ss/As are in G/W1 and G/W4, then fog node1 in layer fog3 will be assigned the monitoring. Cloud as well may monitor the end user specified cases when there is no any fog in the topology that has access to all G/Ws the end user has hired Ss/As from, G/W1 and G/Wn for instance. In addition, and in case of fog node(s) failure, Fog G/W Assignment is in charge of reassigning children fog nodes and/or G/Ws.

The failure plan algorithm is given in algo. 3. If a failure occurred, the failed node id and ids of its children are retrieved (lines 3–5). Lines (6–7) find the nearest fog node in the same layer. If there is no such node or it is far from the geographical location of the failed node, its parent node will be selected (lines 8–9). The reassignment is done for all children (lines 10–11). If previously failed node reconnected, the original branch path will be restored by reassignment of children nodes to the reconnected fog node (lines 13–17).

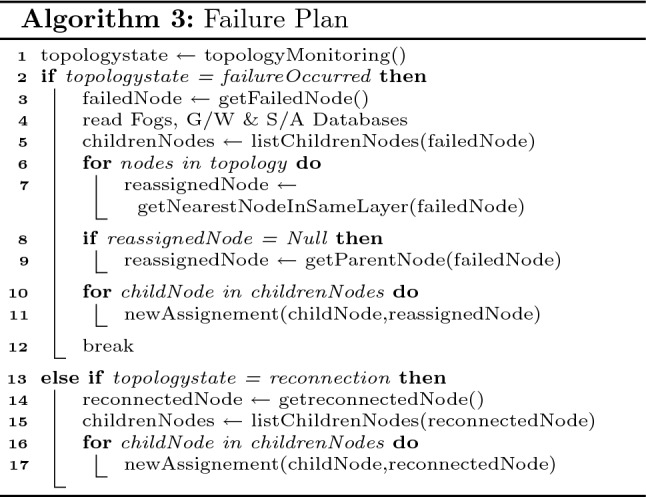



#### Specified cases manager

In some cases, the cloud is the only parent of all branches of G/Ws where the end user sensors/actuators are connected to; for instance, G/W1 and G/Wn in Fig. 1. In such a case, specified cases manager in the cloud is to monitor the specified cases for end users.

#### Fog node manager

All control messages sent from the cloud to fog nodes (e.g. assignments and actuation commands either generated by SCM or received from end users) are sent through Fog Node Manager.

#### Fogs, G/W & S/A database

Being in the cloud, this database stores information about all devices in the entire network; such information is used for S/A physical selection, specified case monitoring, and assignment.

### SALFSD layered fog

**Figure 4:**
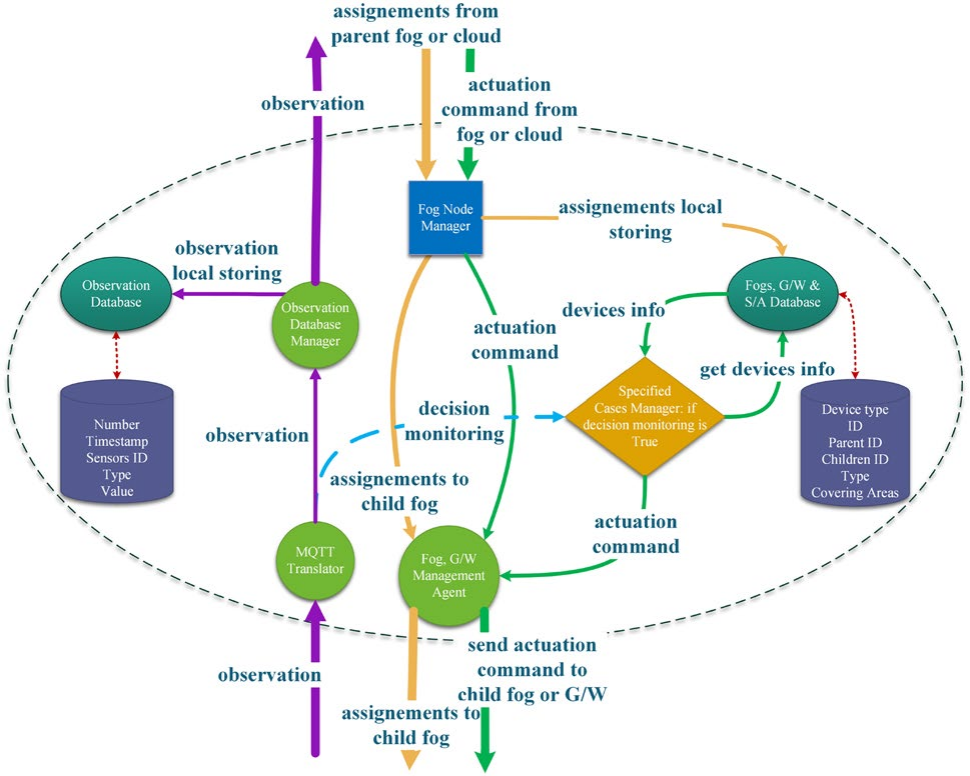
Fog node design with all its components and types of internal messages among its models.

This subsection explains a fog node components, their responsibilities, and communication among them as presented in Fig. 4.

#### Fog node manager

Fog node manager receives control messages from the cloud or from parent fog node. Two types of control messages are received: (1) Assignment: Either to be forwarded to child fog node or to the node itself. The former is passed to “Fog, G/W Management Agent” and the later is stored locally. (2) Actuation Request: Either to the current node itself or to its child node, both are passed to “Fog, G/W Management Agent”.

#### Specified cases manager

Specified cases manager in each fog node is to monitor the specified cases for end users whom their hired Ss/As come under it. For example, fog node 2 in fog layer 2 monitors specified cases of end users hired Ss/As in G/W 2 and G/W3, fog node 2 in fog layer 2 monitors for end user of either G/Ws2,3, and G/W4, fog node 1 in fog layer 3 monitors for end users of G/W1 and any of G/Ws2,3, and 4.

The above explained monitoring is the default setup by the cloud as per SALFSD infrastructure and end users hired Ss/As. However, to avoid increased actuation latency, if a particular fog node in any layer is overloaded, it handles the monitoring to its parent node as shown in algo. 4. For each observation, monitoring manager checks if the node has previously offloaded the monitoring to its parent but now it is not overloaded; hence it will retrieve the monitoring from its parent (lines 1–6).

If there was no previous offloading, the fog node makes sure that it has to monitor the observation either being the default monitoring node or its child node has offloaded the monitoring task to it (line 7). If so, the fog node checks its monitoring load. If the fog node is overloaded, it offloads the monitoring to its parent node (lines 9–12); otherwise, it will go ahead with checking the SCM (lines 13–15). In case SCM generates an actuation command, it is passed to (Fog, G/W Management Agent).
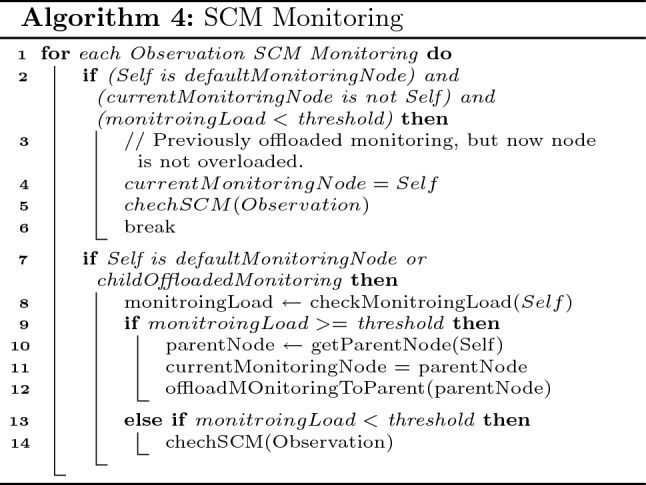


#### Fog, G/W management agent

Fog, G/W Management Agent receives two types of messages: (1) Actuation commands either from Fog Node Manager or from Specified Cases Manager. In any case, if the current fog node is in the lower fog layer, the command is passed to the designated G/W where the targeted actuator is connected to. Otherwise, the actuation command is forwarded to the designated child fog node. (2) Assignment from Fog Node manager. In this case, the current fog node is clearly not in lower fog layer; hence it forwards the assignment to the designated child fog node.

#### MQTT translator

Message Queuing Telemetry Transport (MQTT)^9^ is a light weight messaging transport protocol. designed to be suitable for IoT devices and machine to machine communication. However, MQTT does not have designated message form; hence SALFSD uses a translator for avoiding any mixups in the sensors’ observations, sensors’ channels, ids, time-tamp etc.

MQTT translator is the fog node entry for incoming observations for gateways. Upon receiving each message from gateways, the translator first checks the observation and drop it if it is found to be corrupted algo 5. (lines 1–2.) Otherwise, only observation value and required information in the message will be extracted, and a copy is sent to both specified cases manager and observation database manager; algo 5. (lines 3–4).
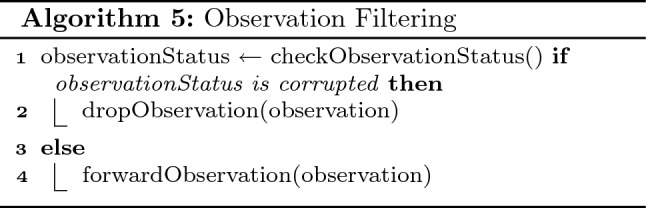


#### Observation database manager

Observation Database Manager forwards all observations received from MQTT Translator to the parent (fog or the cloud) and stores a copy locally.

#### Fogs, G/W & S/A database

Being in fog node, this database stores information about all devices that comes under it in the network topology. This information is used for assignment.

### Gateway in SALFSD

**Figure 5:**
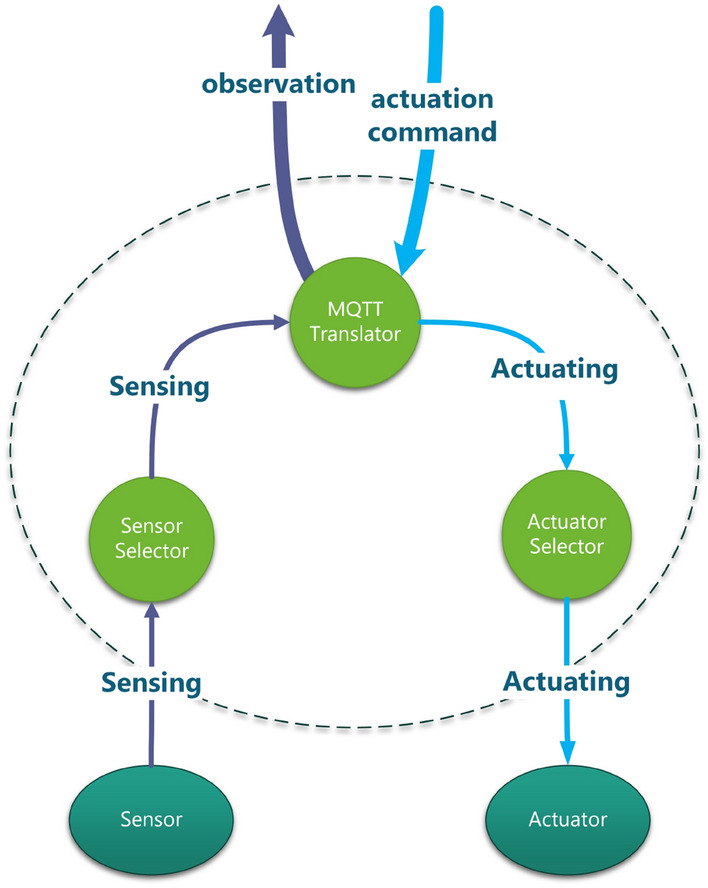
Gateway design with all its components and types of internal messages among its models.

Gateways in SALFSD are considered dumb gateways as they do not do any form of data processing; only forward observations from sensors to fog node and apply the actuation commands received from fog nodes on the sensors. Below subsections explain SALFSD gateway components and their responsibilities as depicted in Fig. 5.

#### MQTT translator

MQTT Translator is responsible for communication between G/W and fog node. Once it receives actuation command messages, it passes only the necessary information to the Actuator Selector; Actuator cloud id, actuation period ( alarm actuator for instance.) Also, observations received from Sensors Selector are sent to the fog node via the dedicated MQTT channel.

#### Actuator selector

Actuator Selector receives actuation commands from MQTT Translator and sets the commands in the designated channel of the target physical actuator.

#### Sensor selector

Sensor Selector receives sensing from the physical sensor, bends them with the correct sensor cloud id and pass it to MQTT Translator.

## Experiment methodology and results discussion

To evaluate the contributions in this work, we have simulated the architecture using YAFS (Yet Another Fog Simulator)^10^. This section explains each simulation type, modes and compares the results.

### YAFS

YAFS is a discrete event simulator very similar to iFogSim yet more flexible. It provides powerful tools to easily design fog computing applications, implementing routing strategies, and also permit a dynamic resources allocation and topology management, which are very useful to implement failure plan scenarios. YAFS is build using two main Python libraries. (1) Simpy for discrete event simulation, it represents the core of the simulator which control the different process of the simulation such as the tasks generation, messages transmission and execution. (2) NetworkX is the graph theory library integrated into YAFS to define system architecture in which nodes represent the topology device such as sensors, actuators, fog devices, and the cloud, along with edges which represent the link among system devices.

### Experiments

Before we explain the experiments, we show the general simulation setup parameters for all experiments in Table 2 and the equations used to calculate latencies with their abbreviations in Table 3.

**Table 2:** General simulation setup parameters.

Parameter	Value
Cloud CPU	16 Ghz
Fog CPU	2 to 6 Ghz
Observation/actuation message instructions	1 Million instructions
Observation/actuation message size	10 KByte
Bandwidth	2 to 6 Mbyte
Number of fog layers	3
Number of fog nodes	39
Simulation time	500

**Table 3:** Abbreviations used in the equations.

Abbreviation	Definition
m	Message
n	Computing node
LCP	Computing latency
LCN	Communication latency

Computing latency (LCP):

6 $$\begin{aligned} LCP_m = \frac{instructions_{m}}{CPU_{n}} \end{aligned}$$

*LCP actuation messages:*

7 $$\begin{aligned} LCP_{actuation} = \sum _{0}^{i} LCP_{m} (actuation) \end{aligned}$$

*LCP observation messages:*

8 $$\begin{aligned} LCP_{observation} = \sum _{0}^{i} LCP_{m} (observation) \end{aligned}$$

Communication latency (LCN):

9 $$\begin{aligned} LCN_m = \frac{size_{m}}{bandwidth} \end{aligned}$$

*LCN actuation messages:*

10 $$\begin{aligned} LCN_{actuation} = \sum _{0}^{i} LCN_{m} (actuation) \end{aligned}$$

*LCN observation messages:*

11 $$\begin{aligned} LCN_{observation} = \sum _{0}^{i} LCN_{m} (observation) \end{aligned}$$

The different types of experiments, their configuration and results are discussed below.

#### Failure plan

As every fog node is known to be a point of failure, failure plan is to monitor the failure of any fog node and reassign its tasks to the nearest fog node in the same level or its parent fog node. Here we evaluate the benefit of having reassignment by conducting and comparing average sent and received observation and actuation commands for the following modes of tests with regards to failure plan: Mode 1, simulation runs without failing any fog node. Mode 2, ten fog nodes are randomly failed during simulation without reassignment. Mode 3, we randomly fail ten fog nodes and reassign their tasks to other fog nodes. Simulation configuration parameters of failure plan are specified in Table 4.

**Table 4:** Configuration parameters for failure plan modes.

Parameter	Value
General parameters for all tests	
Failure mode	1,2, and 3
Number of failed fog nodes	10, for modes 2 and 3 only
Actuation mode	Both end user and SCM
Monitoring mode	No offloading
Corrupted messages mode	No corrupted messages
Number of areas per user	Random
For 100 users tests with 80 gateways	
Number of sensors and actuators	237 sensors, 240 actuators
For 200 users tests with 91 gateways	
Number of sensors and actuators	265 sensors, 251 actuators
For 300 users tests with 74 gateways	
Number of sensors and actuators	234 sensors, 224 actuators
For 400 users tests with 79 gateways	
Number of sensors and actuators	243 sensors, 230 actuators
For 1000 users tests with 73 gateways	
Number of sensors and actuators	201 sensors, 218 actuators

**Figure 6:**
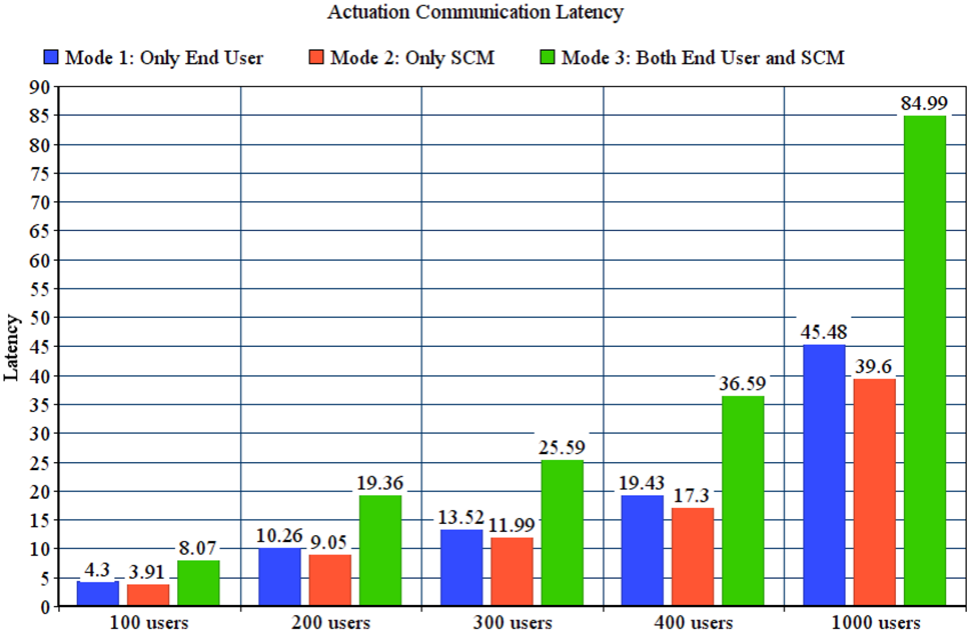
Packets loss due to failed fog nodes.

Figure 6 presents the results of average observation generated by sensors, average observation successfully received by cloud, average actuation requests generated, and average actuation successfully executed; the average is for five tests (i.e. 100, 200, 300, 400, 1000 users). Results show that mode 3 avoids loss of observations and actuation requests compared with mode 2. In case of no reassignment, the network topology remains disconnected; it could have many disconnection points depending on the number of failed fog nodes hence leading to a vast amount of missing messages.

#### Actuation mode

In SAaaS, typically end user gets the observations, analyse them and then decide the proper actuation; if any. However, as explained in “Contribution” section, SALFSD deploys users’ SCM in fog nodes as well as the cloud. We evaluate the benefit of having SCM by conducting and comparing actuation communication latencies for the following modes of tests: Mode 1, actuation request is generated by end users only. Mode 2, actuation request is generated by SCM only. Mode 3, both end users and SCM generate actuation requests. Simulation configuration parameters of actuation modes are specified in Table 5.

**Table 5:** Configuration parameters for actuation modes.

Parameter	Value
General parameters for all tests	
Failure mode	Failure with reassignment
Number of failed fog nodes	10
Actuation mode	1, 2 and 3
Monitoring mode	No offloading
Corrupted messages mode	No corrupted messages
Number of areas per user	Random
For 100 users tests with 79 gateways	
Number of sensors and actuators	395 sensors, 226 actuators
For 200 users tests with 70 gateways	
Number of sensors and actuators	350 sensors, 195 actuators
For 300 users tests with 90 gateways	
Number of sensors and actuators	450 sensors, 257 actuators
For 400 users tests with 70 gateways	
Number of sensors and actuators	350 sensors, 217 actuators
For 1000 users tests with 73 gateways	
Number of sensors and actuators	750 sensors, 237 actuators

**Figure 7:**
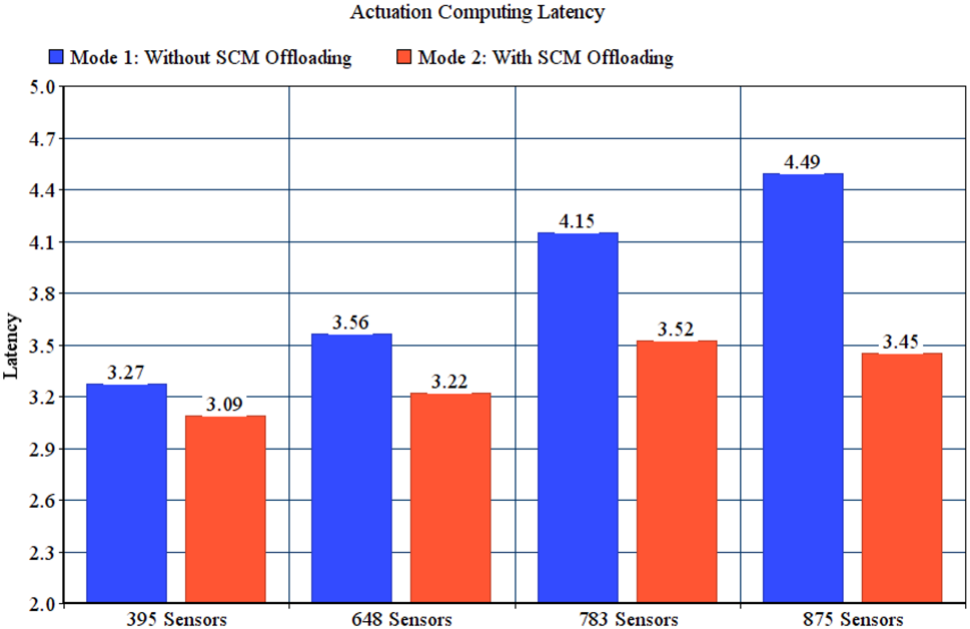
Actuation communication latency for actuation modes.

Figure 7 shows actuation communication latency. In mode 1 and 3, the observations have to travel to the cloud then to the end user. Then the end user will analyse them and decide the proper actuation to be taken and sends back the actuation command; this takes longer time compared to mode 2. Mode 2 always results in less communication latency, which makes actuation commands generated by SCM in several network points fits time-sensitive applications like health care.

#### Monitoring offloading

As explained in “Cloud gateway” section, SCM may be offloaded to parent fog node in case of the fog node responsible for monitoring is overloaded. To evaluate the benefit of offloading SCM we compared actuation computing latencies for two test modes: Mode 1, no offloading; fog node will keep monitoring even if it is overloaded. Mode 2, overloaded fog node offloads SCM to its parent. Simulation configuration parameters of monitoring offloading modes are specified in Table 6.

**Table 6:** Configuration parameters for SCM monitoring offloading modes.

Parameter	Value
General parameters for all tests	
Failure mode	Failure with reassignment
Number of failed fog nodes	10
Actuation mode	Both end user and SCM
Monitoring mode	No offloading
Corrupted messages mode	No corrupted messages
Number of users	100
Number of areas per user	Random
For 395 sensors tests with 79 gateways	
Number of actuators	226
For 648 sensors tests with 81 gateways	
Number of actuators	254
For 783 sensors tests with 77 gateways	
Number of actuators	230
For 875 sensors tests with 87 gateways	
Number of actuators	255

**Figure 8:**
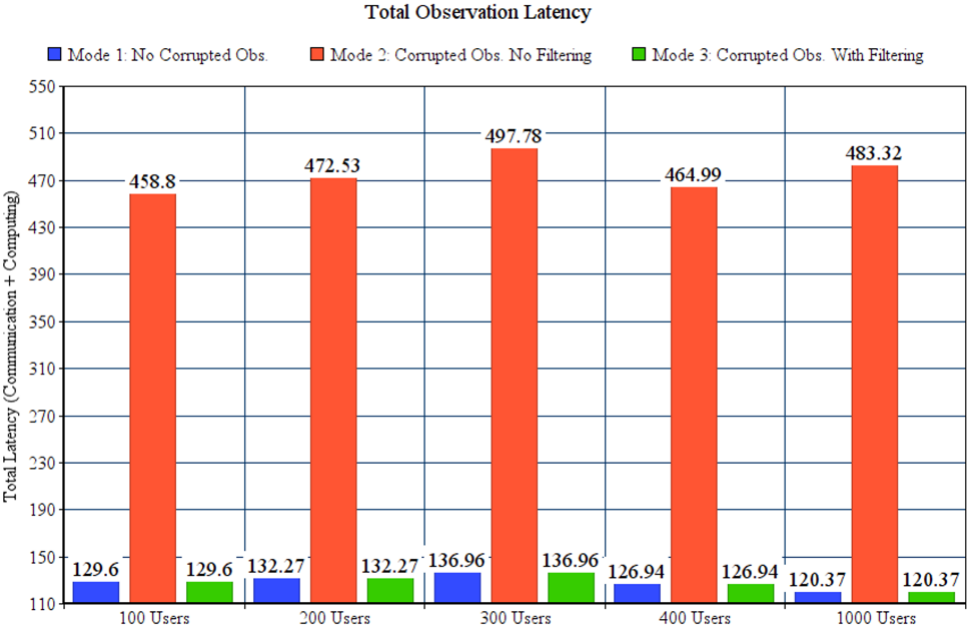
Actuation computing latency for SCM monitoring offloading modes.

Figure 8 shows that offloading reduces the actuation computing latency in all conducted tests. Without offloading, monitoring observations is delayed as the node is overloaded, and hence the generation of actuation command.

#### Corrupted observation filtring

As explained in “Cloud gateway” section, MQTT translator filters all incoming observations and drops the corrupted once, if any. To evaluate the benefit of such filtring, we have compared total observation latencies (communication latency + computing latency) for three test modes: Mode 1, no corrupted observations are generated. Mode 2, corrupted observations are generated and also forwarded. Mode 3, MQTT translator filter and drops the corrupted observations. Only uncorrupted observations are forwarded. Simulation configuration parameters of observation filtering modes are listed in Table 7.

**Table 7:** Configuration parameters for observation filtring modes.

Parameter	Value
General parameters for all tests	
Failure mode	Failure with reassignment
Number of gateways	81
Number of failed fog nodes	10
Actuation mode	Both end user and SCM
Monitoring mode	No offloading
Corrupted messages mode	1, 2 and 3
Number of areas per user	Random
Parameters for 100 users tests	
Number of sensors and actuators	243 sensors, 234 actuators
Parameters for 200 users tests	
Number of sensors and actuators	246 sensors, 243 actuators
Parameters for 300 users tests	
Number of sensors and actuators	255 Sensors, 254 actuators
Parameters for 400 users tests	
Number of sensors and actuators	242 sensors, 254 actuators
Parameters for 1000 users tests	
Number of sensors and actuators	222 sensors, 242 actuators

**Figure 9:**
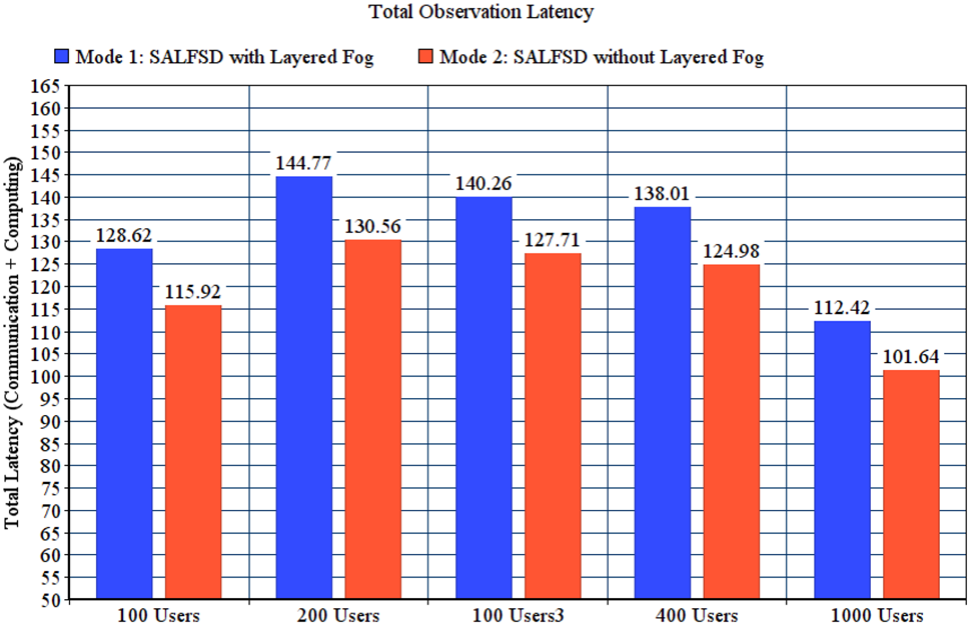
Total observation latency corrupted messages modes.

Figure 9 shows that corrupted observation filtering in mode 3 results in the same total observation latency as mode 1 in all conducted tests.

#### Comparing SALFSD with and without layered fog

To present the value added benefits of layered fogs in SAaaS combined with the cloud, we have compared total observation latency (communication latency + computing latency), actuation computation, and communication latencies for two test modes: Mode 1, SALFSD with layered fogs. Mode 2, SALFSD without layered fogs; only the cloud and gateways with Ss/As. In addition, to keep the same distance between the gateways and the cloud, we have placed routers nodes (instead of fog nodes for the same number of layers in mode 1; i.e. 3 layers.) Simulation configuration parameters are listed in Table 8.

**Table 8:** Configuration parameters for layered fogs modes.

Parameter	Value
General parameters for all tests	
Failure mode	No failure
Actuation mode	Both end user and SCM
Monitoring mode	No offloading
Corrupted messages mode	No corrupted messages
Number of areas per user	Random
For 100 users tests with 80 gateways	
Number of sensors and actuators	237 sensors, 240 actuators
For 200 users tests with 91 gateways	
Number of sensors and actuators	265 sensors, 251 actuators
For 300 users tests with 74 gateways	
Number of sensors and actuators	234 sensors, 224 actuators
For 400 users tests with 79 gateways	
Number of sensors and actuators	243 sensors, 230 actuators
For 1000 users tests with 73 gateways	
Number of sensors and actuators	201 sensors, 218 actuators

**Figure 10:**
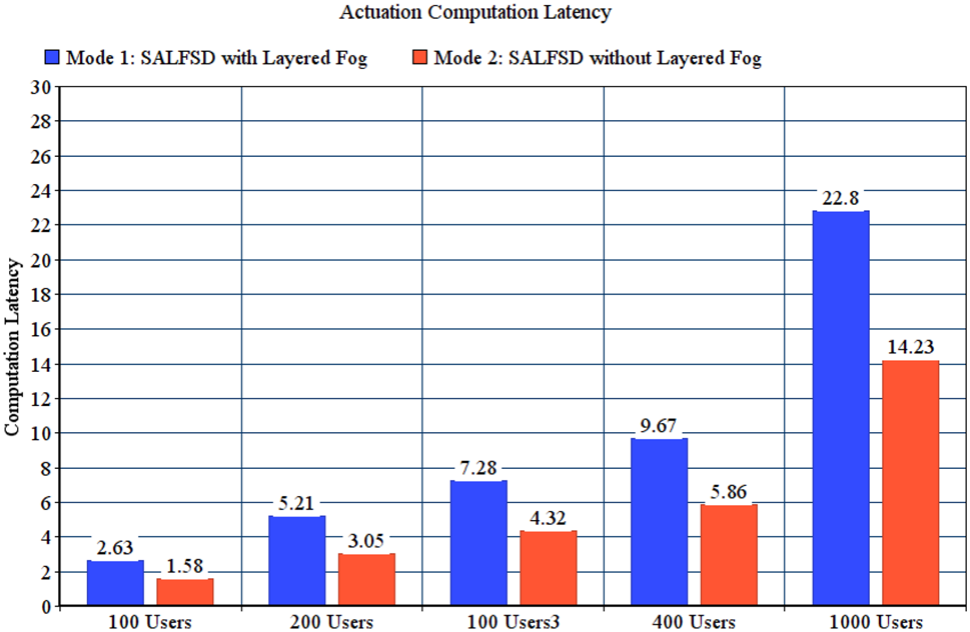
Total observation latency, SALFSD with and without layered fog.

**Figure 11:**
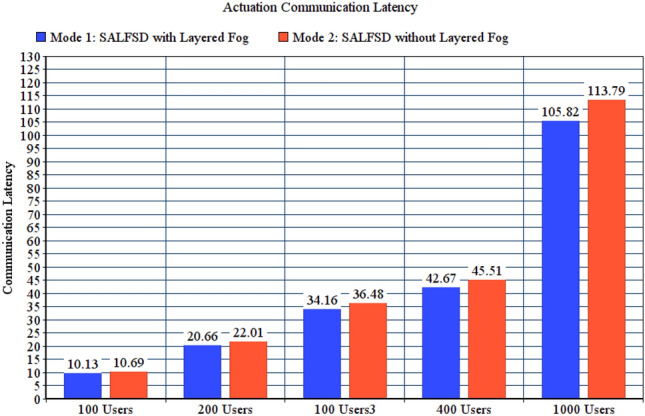
Actuation computation latency, SALFSD with and without layered fog.

**Figure 12:**
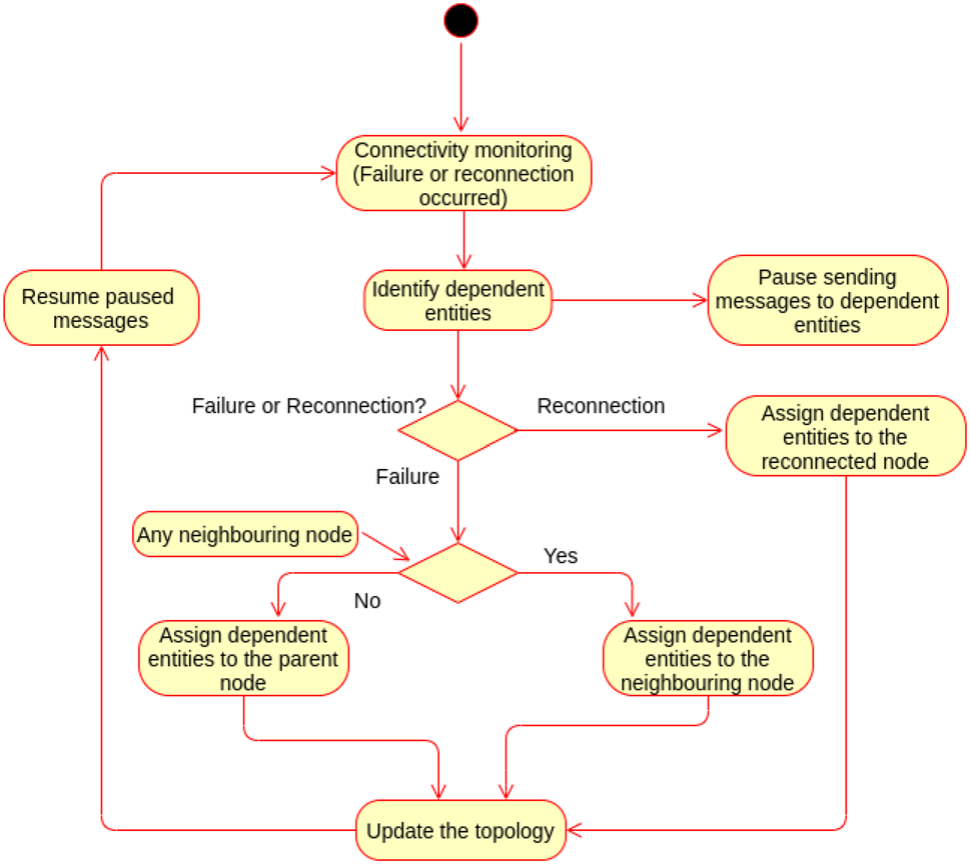
Actuation communication latency, SALFSD with and without layered fog.

Figure 10 shows total observation latency for both modes. Despite having the same distance between the cloud and gateways which makes the same communication latency, the reason that makes mode 1 results in increased total observation latency is having more computing in the layered fog; Fog nodes process the observation for the sake of SCM. The same is also applied to actuation computing latency presented in Fig. 11; which are considered weak points in SALFSD. However, the trade-off is that mode 1 results in less actuation communication latency; as presented in Fig. 12.

### General discussion on the results

The added layered fog to SAaaS basic design and the proposed features implemented inside fog nodes as well as the cloud has been simulated and evaluated in terms of the following metrics: (1) total delivered messages, (2) computing and communication latencies for observation and actuation messages. The simulation was conducted in the same environment with different scenarios regarding the number of end users, gateways, Ss/As in IoT layer, and areas per end user from which their Ss/As are selected.

The overall study of the results shows that besides keeping the infrastructure connected, failure reassignment prevents loss of observation and actuation messages, consequently resulting in 100 per cent messages delivery.

Also, results show that deploying end users SCM in the cloud and fog nodes generally decreases the actuation latency as the decision of triggering the actuation command is being taken closer to IoT layer. Furthermore, SCM offloading also prevents increased actuation latency in case fog nodes are overloaded due to receiving a vast amount of observation to be monitored (an IoT associated issue; i.e. Big Data^11^). Such a case is expected with the increased number of IoT devices in SAaaS paradigm. This is significant in time-sensitive applications.

In addition, dropping the corrupted observations reduces the unnecessary consumption of network bandwidth. Corrupted observation may be sent from sensors, or being effected while forwarding by fog node. The former is dropped by the fog node connected to the gateway in the lower fog layer. The later is dropped by parent fog node in upper fog layers.

Comparing SAaaS with and without layered fog presented less actuation communication latency with layered fog. Adding this point to the benefits of features implemented inside fog nodes, totally shows the performance enhancement provided by SALFSD to the basic SAaaS architecture.

## Formal verification of architecture correctness

This section proves the correctness of the failure plan and topology connectivity monitoring, SCM offloading, and observation filtering. First, the related architecture invariants and properties are listed below.

### Architecture invariants and properties

(I) Architecture invariants:
  1. The architecture is fully connected if all branches are connected.
  2. Failure of any fog node in a branch leads to the branch being disconnected, hence the architecture is partially disconnected.
  3. If a branch is disconnected, the dependent entities (Children fog nodes of the failed fog node, Gateways, and IoT devices) are unreachable.
(II) Final objective:
  4. Reassignment of dependent entities of the failed node to any other node reconnects the branch and make the dependent entities reachable again, and hence the architect is fully connected again.
(III) Functional properties:
  5. When failure occurs, all the dependent entities are eventually reachable either through a neighbouring node of the failed fog node or through its parent node.
  6. Any failed fog node may not always reconnect, hence the original branch path may not always be restored.
  7. When a failure occurs, sending messages to dependent entities of the failed fog node is paused until an alternative path is established by the reassignment.
  8. The reassignment can always end correctly either to a neighbouring node or to the parent node.

### Connectivity monitoring and failure plan proof

This subsection proves the correctness of the failure plan and topology connectivity monitoring.

Let T represents the topology.

FN Represents the set of *N* number of fog nodes in the topology.

*fxi* is a particular fog node *i* such that

*fxi* $$\in \{fx1, fx2, fx3,\ldots \ldots , fxN\}$$

Fully connected (Fc) Represents the topology when all its branches are connected.

Partially disconnected (Pd) Represent the topology when any fog node fails resulting on a branch disconnection.

Virtually partially disconnected (Vpd) Represent the topology when any previously failed node reconnects, this is a temporally state being made to pause sending messages to dependent entities and do the reassignment to the connected fog node so that the original path of the branch is restored.

The following scenarios are realised for the failure plan:

- Scenario (1): A fog node fails, and there is no any neighbouring fog node in the same layer and same location.
  - The system topology T is working in the normal state; Fc. Then a fog node *fxi* fails. The connectivity monitoring discovers such node failure and starts the failure processing. At this moment, the topology has lost one branch; hence the topology is in Pd due to *fxi* failure. The dependent entities (children fog nodes, gateways, and sensors and actuators) on *fxi* are identified, then sending messages to such entities is temporally paused. Then topology will be searched for the nearest neighbouring fog node in the same layer and same location of *fxi*; there is no such node. Hence the dependent entities will be assigned to the parent node of *fxi*. The topology is then updated to reflect the new assignment and reconnect the branch, changing the topology states to Fc. The paused messages are now resumed.
- Scenario (2): A fog node fails, and there is a neighbouring fog node in the same layer and same location.
  - The system topology T is working in the normal state; Fc. Then a fog node *fxj* fails. The connectivity monitoring discovers such node failure and starts the failure processing. At this moment, the topology has lost one branch; hence the topology is in Pd due to *fxj* failure. The dependent entities on *fxj* are identified, then sending messages to such entities is temporally paused. Then topology will be searched for the nearest neighbouring fog node in the same layer and same location of *fxj*; such node is found say *fxj+1*. Hence the dependent entities will be assigned to *fxj+1*. The topology is then updated to reflect the new assignment and reconnect the branch, changing the topology states to Fc. The paused messages are now resumed.
- Scenario (3): A previously failed fog node reconnected.
  - The system topology T is working in the normal state; Fc. Then a previously failed fog node *fxi* reconnects. The connectivity monitoring discovers such node reconnection and starts restoring the original path of *fxi* branch. *fxi* depending entities are identified and sending messages to such entities is temporally paused, making the topology to be in Vpd while the original path is restored. The topology is then updated to reflect the new assignment and reconnect the branch, changing the topology states to Fc. The paused messages are now resumed.

**Figure 13:**
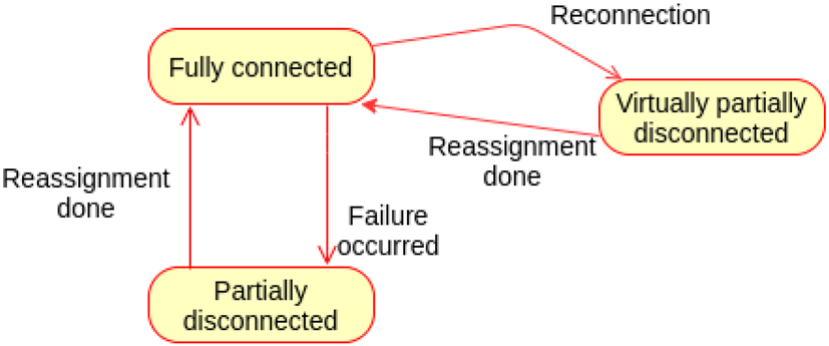
Behaviour of connectivity monitoring and failure plan.

The above scenarios show that failure plan always succeeds in reconnecting the affected branch due to fog node failure, and the final objective is always reached. Figure 13 describes the behaviour of the proposed failure plan.

**State transition**

**Figure 14:**
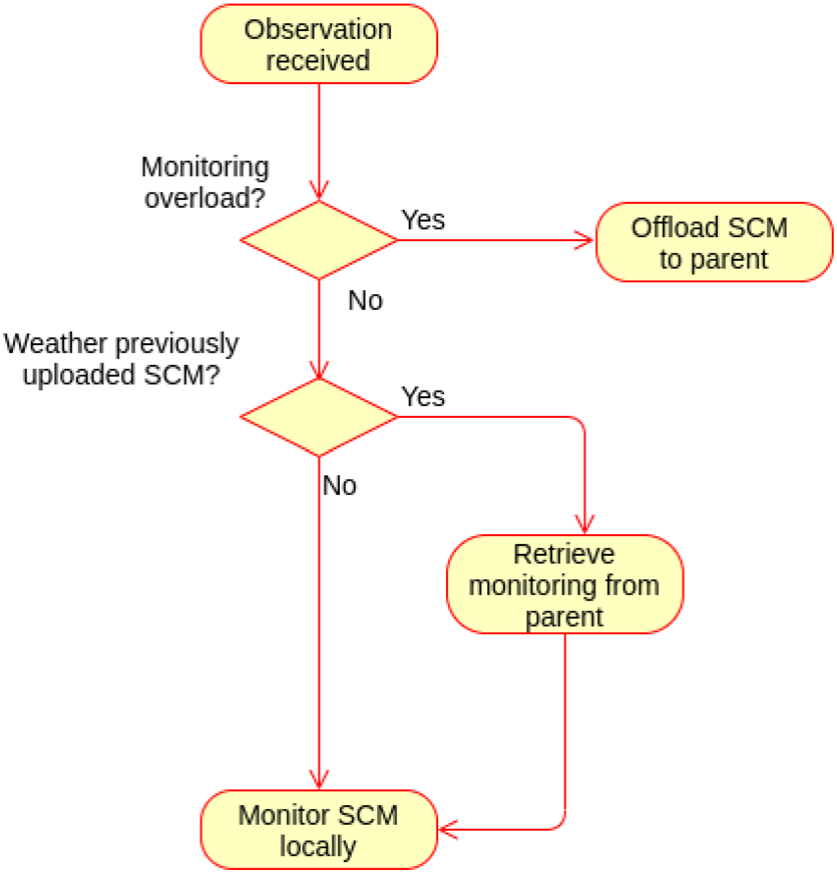
State machine of connectivity monitoring and failure plan.

The state of the topology is said to be fully connected if all its branches are connected, a normal state. Failure of any fog node makes the topology enters the partially disconnected state. Reconnection of any previously disconnected fog node makes the topology temporally enters virtually partially disconnected state. Figure 14 shows the state machine of the proposed failure plan.

The state machine M of the topology is represented as a pentuple

$$\begin{aligned} \hbox {M} = (\hbox {Q}, \varSigma , \delta , q_0, \hbox {F}) \end{aligned}$$

where Q Represents set of sates.

- $\varSigma$ represents the set of inputs needed for transitions.
- $\delta$ represents the transition function.
- $q_0$ represents the initial state.
- F represents the final state.
- $\hbox {Q} =$ { Fully connected, Partially disconnected, Virtually partially disconnected}.
- $\varSigma =$ {Failure occurred (Fo), Reconnection (R), Reassignment done (Rd)}.
- $$q_0 =$$ {Fully connected}.
- $$\hbox {F} =$$ {Fully connected}.

The transitions of the state machines are defined as:

- $\delta$ (Fully connected, Fo) $=$ Partially disconnected.
- $\delta$ (Partially disconnected, Rd) $=$ Fully connected.
- $\delta$ (Fully connected, R) $=$ Virtually partially disconnected.
- $\delta$ (Virtually partially disconnected, Rd) $=$ Fully connected.

The following cases describe the correctness of the connectivity monitoring and failure plan of the architecture topology.

- Case (1): A fog node fails and dependent entities will be assigned to other fog node.
  - $\delta$ (Fully connected, Fo) $=$ Partially disconnected.
  - $\delta$ (Partially disconnected, Rd) $=$ Fully connected $\in$ F, hence accepted.

The topology T state is changed from Fully connected to Partially disconnected (Failure occurred). When reassignment is successfully done (Rd), it enters into the Fully connected state which is the accepted final state in the state machine M. The state transitions are same whether the assignment will be for a neighbouring node or to the parent node of the failed one; hence this case is applied for both scenarios.

- Case (2): a previously failed node reconnected.
  - $\delta$ (Fully connected, R) $=$ Virtually partially disconnected.
  - $\delta$ (Virtually partially disconnected, Rd) $=$ Fully connected $\in$ F, hence accepted.

The topology T state is changed from Fully connected to Virtually partially disconnected (Reconnection). When reassignment is successfully done (Rd) to restore the original branch path, it enters into the Fully connected state which is again the accepted final state in the state machine M.

### SCM monitoring and offloading proof

As explained earlier, SCM is assigned by the cloud to the lowermost fog node connected to the gateway(s) from which the end user Ss/As are connected to. However, this monitoring node can offload the monitoring to its parent if it is overloaded to reduce the actuation commands latency. Later, if the fog node is not overloaded, it can retrieve the monitoring offloaded to its parent.

**Figure 15:**
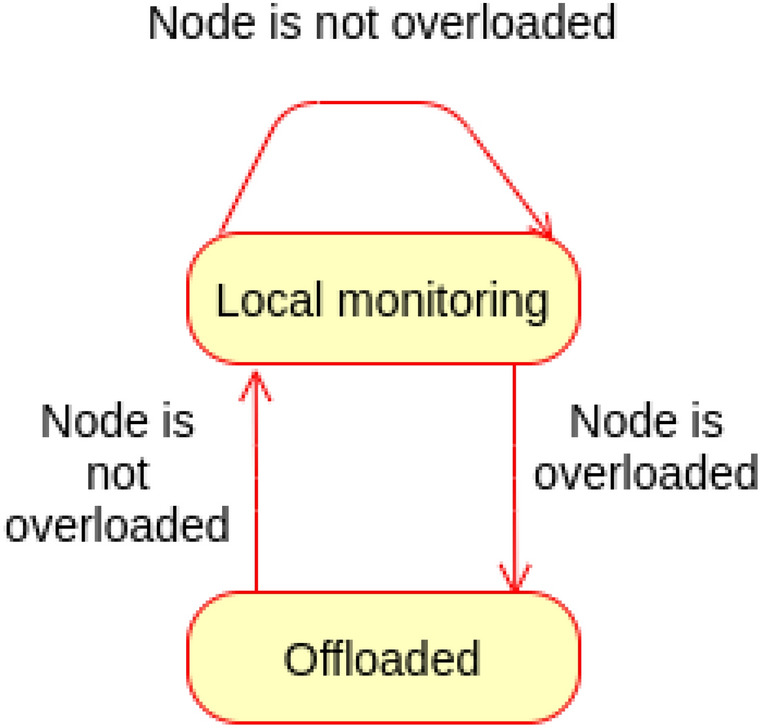
Behaviour of SCM monitoring and offloading.

The following scenarios are realised for the SCM monitoring and offloading, as depicted in Fig. 15:

- Scenario (1): A fog node is not overload; hence it will monitor the SCM locally as assigned by the cloud.
  - For any observation sent to SCM, the specified cases manager will check if the node monitoring load is equal to or more than the threshold. If not, the node will continue monitoring the specified cases assigned to it locally. Also, if the node is not overloaded, it checks whether it has previously offloaded the monitoring to its parent. If so, it will retrieve monitoring from parent and continue monitoring locally.

Note that the threshold may be different from node to node depending on the node processing capability.

- Scenario (2): Fog node is overloaded.
  - If the specified cases manager receives observation during which the node is overloaded (node monitoring load is equal to or more than the threshold), it will offload the monitoring to the parent fog node.

**State transition**

**Figure 16:**
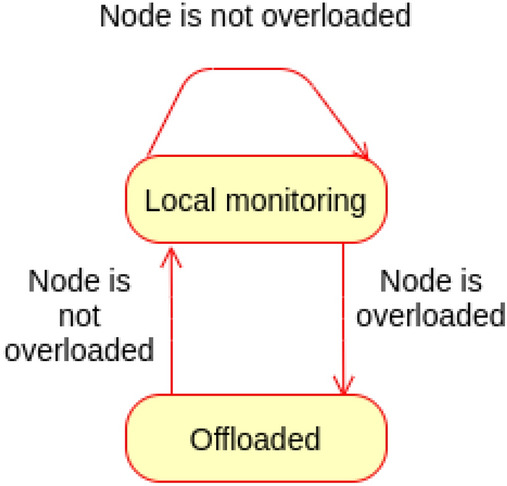
State machine of SCM monitoring and offloading.

The state of SCM monitoring can be either local monitoring or offloaded; this is governed by the threshold of the node monitoring load. Figure  16 shows the state machine of the proposed SCM monitoring and offloading.

The state machine M of SCM monitoring is represented as a pentuple

$$\begin{aligned} \hbox {M} = (\hbox {Q}, \varSigma , \delta , q_0, \hbox {F}) \end{aligned}$$

where Q Represents set of sates.

- $\varSigma$ represents the set of inputs needed for transitions.
- $\delta$ represents the transition function.
- $q_0$ represents the initial state.
- F represents the final state.
- $\hbox {Q} =$ { Local monitoring, Offloaded}.
- $\varSigma =$ {Node is not overloaded (Nno), Node is overloaded (No)}.
- $$q_0 =$$ {Local monitoring}.
- $\hbox {F} =$ {Local monitoring, Offloaded}.

The transitions of the state machines are defined as:

- $\delta$ (Local monitoring, Nno) $=$ Local monitoring.
- $\delta$ ((Local monitoring, No) $=$ Offloaded.
- $\delta$ (Offloaded, Nno) $=$ Local monitoring.

The following cases describe the correctness of the specified cases manager for monitoring and offloading.

- Case (1): The node monitoring load is less than the threshold; the node is not overloaded.
  - $\delta$ (Local monitoring, Nno) $=$ Local monitoring.

The state of monitoring will continue to Local monitoring as long as the monitoring load is less that the threshold. This is an accepted state; $\in$ F.

- Case (2): The monitoring load goes equal or more that the threshold. Therefore the manager will offload the monitoring to the parent node.
  - $\delta$ (Local monitoring, No) $=$ Offloaded.

As the node is overloaded, the monitoring will enter an Offloaded state which goes on as long as the node is load overloaded. This state is also accepted; $\in$ F. This way the monitoring will continue even at the parent node.

- Case(3): The manager has previously offloaded monitoring, but now the node is not overload.
  - $\delta$ (Offloaded, Nno) $=$ Local monitoring.

Once the node is not overloaded, the manager will retrieve the monitoring from the parent node as assigned by the cloud. This is again the same accepted state; $\in$ F.

### Observation filtering proof

**Figure 17:**
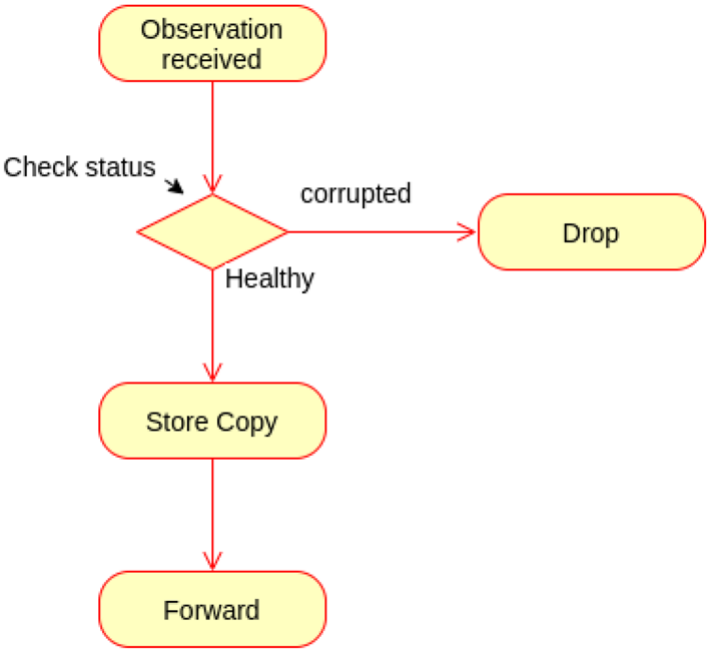
Behaviour of observation filtering.

The observation received to MQTT will be filtered, and only the healthy once will be farther forwarded upward. Figure 17 describes the behaviour of the proposed observation filtering.

The following scenarios are realised for the observation filtering.

- Scenario (1): Received observation is corrupted.
  - Upon receiving any observation, MQTT translator checks its state. If it is corrupted, it will be dropped.
- Scenario (2): If the received observation is not corrupted, a copy will be stored in the fog observation database, and a copy will be forwarded upward.

**State transition**

**Figure 18:**
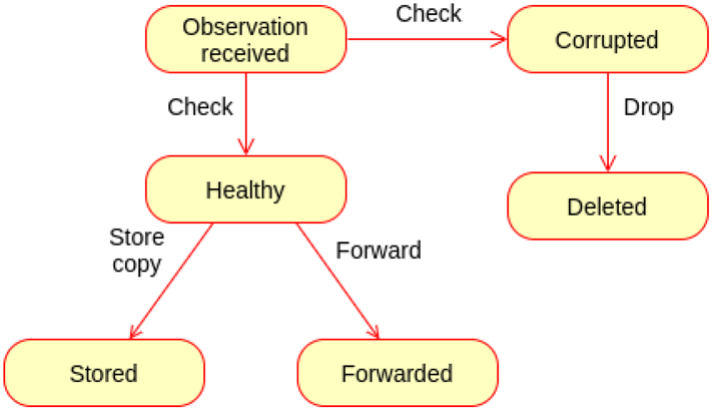
State machine of observation filtering.

The state of observation and the transitions of states are depicted in Fig. 18.

The state machine M of observation filtering is represented as a pentuple

$$\begin{aligned} \hbox {M} = (\hbox {Q}, \varSigma , \delta , q_0, \hbox {F}) \end{aligned}$$

where Q Represents set of sates.

- $\varSigma$ represents the set of inputs needed for transitions.
- $\delta$ represents the transition function.
- $q_0$ represents the initial state.
- F represents the final state.
- $\hbox {Q} =$ {Observation received , Corrupted, Healthy, Stored, Forwarded, Deleted}.
- $\varSigma =$ {Check (Ck), Store copy (Sc), Forward (F), Delete (D)}.
- $$q_0 =$$ {Observation received}.
- $\hbox {F} =$ {Stored, Forwarded, Deleted}.

The transitions of the state machines are defined as:

- $\delta$ (Observation received, Ck) $=$ Healthy.
- $\delta$ (Observation received, Ck) $=$ Corrupted.
- $\delta$ (Healthy, Sc) $=$ Stored.
- $$\delta$$ (Healthy, F) $$=$$ Forwarded.
- $$\delta$$ (Corrupted, D) $$=$$ Deleted.

The following cases describe the correctness of observation filtering.

- Case (1): The received observation is corrupted.
  - $\delta$ (Observation received, Ck) $=$ Corrupted.
  - $$\delta$$ (Corrupted, D) $$=$$ Deleted.

If the received observation is found to be corrupted, it moves into a Corrupted state result of checking. Then it ends in the Deleted state as a final accepted state; $\in$ F.

- Case (2): The received observation is healthy.
  - $\delta$ (Observation received, Ck) $=$ Healthy.
  - $\delta$ (Healthy, Sc) $=$ Stored.
  - $$\delta$$ (Healthy, F) $$=$$ Forwarded.

**Figure 19:**
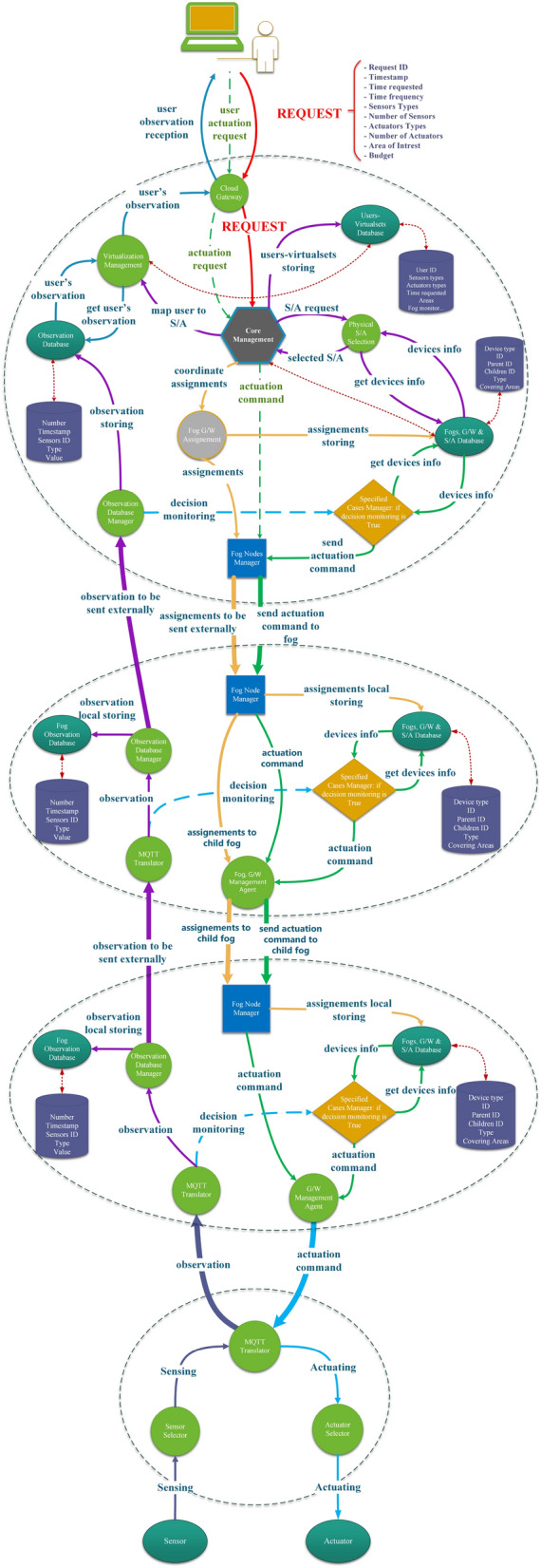
Communication among the cloud, parent fog, child fog, and gateway.

Upon receiving, the observation enters into received sate, as a result of checking it enters into Healthy sate. The Healthy observation must be forwarded to the parent node, and a copy must be stored in the local observation database. Both Stored (locally) and Forwarded (upward) states are accepted as final states of the healthy observation; $\in$ F (Fig. 19).

## Conclusion and future work

Adding scalable and fault resistant layered fog architecture in SAaaS and wisely selecting tasks to be handled by fog nodes has improved the performance of SAaaS. Also, the failure plan suggested in SALFSD provides a persistent availability of the services providers network and hence to its promised services. SALFSD has proved itself in the evaluation results to be a piece of valuable advice for designing SAaaS architectures for IoT applications. This is particularly useful in time-sensitive applications like health care in which any delay due to failure, communication, or computation is critical.

As future work, we are planning to enhance the current work by implementing the reassignment in fog nodes also beside the cloud to make a distributed failure plan such that any fog node can issue the reassignment for any failed node beneath it.
